# Melanoma in Adolescents and Young Adults (AYAs): An Italian Multi-Centric Retrospective Experience

**DOI:** 10.3390/jcm13216445

**Published:** 2024-10-28

**Authors:** Mario Valenti, Andrea D’Arino, Enrico Melis, Andrea Carugno, Paolo Sena, Pasquale Frascione, Carlo Cota, Francesco Piscazzi, Santo Raffaele Mercuri, Vincenzo Russo, Giuseppe Damiano, Alessandra Bulotta, Angelo Valerio Marzano, Maria Alessandra Mattioli, Riccardo G Borroni, Alessandra Narcisi, Antonio Costanzo, Marco Ardigò, Giovanni Paolino

**Affiliations:** 1 Dermatology Unit, IRCCS Humanitas Research Hospital, 20089 Rozzano, Italy; mario.valenti@hunimed.eu (M.V.); marco.ardigo@hunimed.eu (M.A.); 2 Department of Biomedical Sciences, Humanitas University, Pieve Emanuele, 20089 Milan, Italy; 3 UOSD Dermatologia Oncologica, San Gallicano Dermatological Institute IRCCS, 00144 Rome, Italy; 4 Dermatology Unit, Department of Medicine and Surgery, University of Insubria, 21100 Varese, Italy; 5 Dermatology Unit, ASST Papa Giovanni XXIII, 21100 Bergamo, Italy; 6 UOSD Dermatopatologia e Biologia Molecolare ad Indirizzo Dermatologico, San Gallicano Dermatological Institute IRCCS, 00144 Rome, Italy; 7 Unit of Dermatology IRCCS Ospedale San Raffaele, 20132 Milan, Italypaolino.giovanni@hsr.it (G.P.); 8 Unit of Immuno-Biotherapy of Melanoma and Solid Tumors, IRCCS Ospedale San Raffaele, 20132 Milan, Italy; 9 UO Oncologia Medica, IRCCS Ospedale San Raffaele, 20132 Milan, Italy; 10 Dermatology Unit, Fondazione IRCCS Ca’ Granda Ospedale Maggiore Policlinico, 20122 Milan, Italy; 11 Department of Pathophysiology and Transplantation, Università Degli Studi di Milano, 20122 Milan, Italy

**Keywords:** melanoma, skin cancer, AYA, adolescent and young adult oncology, adolescent and young adult melanoma, melanoma localization, Breslow thickness, melanoma ulceration, sentinel lymph node biopsy, survival

## Abstract

**Background:** Melanoma is currently the most prevalent malignant neoplasm among adults and represents the second most common cancer in both sexes among individuals aged 0 to 39 years. This retrospective multicenter study delves into the distinctive clinical, anamnestic, histopathologic, and prognostic attributes of melanoma in Adolescent and Young Adults (AYA), defined as patients diagnosed at ≤40 years, across four Italian centers. **Methods:** Through a computer-based review of clinical records from 1 January 2010 to 30 September 2023, AYA melanomas were contrasted with non-AYA melanomas (>40 years) among 1452 patients. Data on demographics, melanoma localization, histological type, Breslow thickness, ulceration, and sentinel lymph node (SLN) biopsy status were meticulously collected and analyzed. **Results:** Our analysis revealed a female predominance in the AYA group and a male predominance in the non-AYA group, with significant differences in anatomical localization and histological types between the two. AYA melanomas showed nearly equal trunk and limb involvement, contrasting with the trunk predominance in non-AYA melanomas. While Breslow thickness was similar across both groups, the presence of ulceration and total number of nevi showed no significant difference. Survival analysis indicated a marginally higher Disease-Free Survival (DFS) in AYA patients compared to non-AYA patients, without a significant difference in Overall Survival (OS). **Conclusions:** This study highlights demographic and clinical distinctions between AYA and non-AYA melanoma patients, underscoring the need for tailored follow-up and treatment strategies. Despite these insights, the heterogeneity of melanoma among young adults calls for further research, including genetic analyses, to fully understand this unique melanoma subgroup. Indeed, AYA melanoma patients could represent a different and specific target for both follow-up and treatments.

## 1. Introduction

Malignant melanoma is a highly aggressive malignancy known for its early propensity to metastasize, and its global incidence continues to rise. Despite significant advances in the development of targeted therapies and immunotherapies, the prognosis for patients with advanced-stage melanoma remains generally poor. This is largely due to the tumor’s ability to evade the immune system and its inherent resistance to conventional treatments. While surgical excision is the preferred treatment for early-stage melanoma, as the disease progresses to more advanced stages, systemic therapies become necessary to control the spread of the cancer and improve patient outcomes. Unfortunately, even with these advanced therapies, the five-year survival rate for patients diagnosed with metastatic melanoma remains low, highlighting the aggressive nature of the disease.

One of the most important prognostic factors in melanoma is Breslow thickness, which measures the depth of tumor invasion. Other critical factors include ulceration, vascular or lymphatic invasion, and host immune response. These indicators are critical in determining disease severity and guiding appropriate treatment. The classification system based on tumor size, lymph node involvement and the presence of metastases (TNM staging) remains fundamental for risk stratification, influencing both clinical decisions and therapeutic options. Early detection is vital for improving the survival rates in melanoma patients. This is particularly important for patients in stages II-IV, where adjuvant therapies, including immune checkpoint inhibitors and targeted therapies (e.g., BRAF and MEK inhibitors), are considered post-surgical resection. These treatments have shown promise in delaying disease progression and prolonging survival in patients with high-risk or metastatic melanoma.

Melanoma is currently the most prevalent malignant neoplasm among adults and represents the second most common cancer in both sexes among individuals aged 0 to 39 years [1]. Patients within this age group are classified as Adolescents and Young Adults (AYAs) with melanoma. The increasing prevalence of melanoma among AYAs can be attributed to a multitude of environmental and behavioral factors. One of the most significant contributors to the rising incidence of melanoma among younger populations is the increased use of indoor tanning devices. The utilization of tanning beds prior to the age of 30 has been demonstrated to elevate the risk of melanoma by 75%, thus constituting a significant risk factor for the development of the disease in this age group. Furthermore, elevated exposure to ultraviolet (UV) radiation—often in the absence of sufficient protection, such as sunscreen or protective clothing—also plays a pivotal role in elevating melanoma risk among AYAs. These behaviors, when coupled with the cultural perception of tanned skin as a beauty standard, contribute to the rising incidence in this demographic [2]. Another important factor to consider is the role of improved screening and diagnostic methods. For instance, the widespread use of dermoscopy, a non-invasive technique that allows for the detailed examination of pigmented lesions, has led to earlier detection of melanoma in AYAs.

In light of the rising global incidence of melanoma in young people, it is imperative to gather comprehensive clinical and epidemiological data on AYAs melanoma in order to optimize treatment strategies. Notwithstanding the growing number of diagnoses in this age group, the extant literature on AYAs melanomas remains scarce. A number of studies have sought to elucidate the discrepancies between AYAs and older adults with melanoma, particularly with regard to survival rates and clinical characteristics. However, the findings of these studies are often inconsistent or contradictory [1,3,4]. For example, some research indicates that melanomas diagnosed in children and adolescents tend to exhibit more aggressive features, such as a greater propensity for regional lymph node invasion, higher Breslow thickness, and presentation at a more advanced stage compared to adult melanomas [5,6]. Nevertheless, other studies indicate that younger patients frequently exhibit superior disease-specific survival rates compared to older adults despite these aggressive features [3,5,7].

In addition to differences in clinical presentation, there are notable disparities in the histologic subtypes and anatomical locations of melanomas between AYAs and older adults [3,5,7]. For instance, AYAs are more likely to develop melanomas in intermittently sun-exposed areas, such as the trunk, while older adults tend to present with melanomas in chronically sun-exposed regions, like the head and neck. This distribution is likely influenced by cumulative sun damage over a lifetime in older populations, whereas younger patients may have fewer years of chronic sun exposure but may engage in more sporadic intense UV exposure (e.g., tanning) [8,9].

On a genetic level, melanomas in younger patients are frequently associated with specific mutations, most notably in the BRAF gene. This mutation is present in approximately 40–60% of all melanomas and is especially common in AYAs. In contrast, melanomas in older patients are more frequently associated with mutations in the NRAS and NF-1 genes or mutations related to chronic sun exposure, such as those observed in the TP53 gene [8,9].

In the case of familial melanoma, germline mutations in certain high-risk genes, such as CDKN2A and CDK4, significantly increase the likelihood of developing melanoma, often at a younger age and with a higher propensity for multiple primary melanomas. CDKN2A, in particular, is one of the most well-studied genes associated with familial melanoma and plays a critical role in regulating the cell cycle by encoding proteins p16INK4A and p14ARF, both of which are tumor suppressors. Mutations in CDKN2A are found in approximately 20–40% of familial melanoma cases. Moreover, these patients are also at increased risk for other cancers, particularly pancreatic cancer, which underscores the broader implications of genetic predispositions in melanoma syndromes [8,9].

The term “multiple primary melanomas” (MPM) is used to describe the occurrence of two or more distinct primary melanomas in the same individual. This condition is more frequently observed in patients with genetic predispositions, such as mutations in CDKN2A and CDK4, which are commonly associated with familial melanoma. These individuals are at a markedly elevated risk of developing further melanomas over their lifetime, emphasizing the necessity of ongoing dermatological monitoring. MPMs tend to manifest with a more aggressive clinical course and are often diagnosed at a younger age compared to sporadic melanomas, with an increased incidence in patients who have already been diagnosed with juvenile melanoma [8,9]. The genetic landscape of melanoma is diverse and multifaceted. The presence of multiple primary melanomas and familial cases underscores the need for early genetic screening, especially in younger populations and those with a family history of the disease.

These factors contribute to the lack of specific treatment guidelines for AYAs melanoma, resulting in its management following the same protocols as non-AYAs melanoma. Despite this, it remains unclear whether AYAs melanoma shares the same biological characteristics as adult melanoma. Most likely, the differences in the immune surveillance mechanism could justify any clinic-pathologic and prognostic differences between young and older melanoma patients. However, the current gap in knowledge in AYA patients emphasizes the need for more comprehensive studies focusing on AYAs to develop age-appropriate treatment strategies and improve long-term outcomes in this underserved cohort [3].

The goal of our study is to get insights into the clinical, anamnestic, histopathologic, and prognostic characteristics of AYAs melanoma patients treated at four Italian Tertiary Referral Centers. By closely examining this unique patient population, we aim to expand the existing knowledge on the challenges and needs of AYAs with melanoma. It is expected that the current research will further contribute to the development of more effective and personalized methods for diagnosing and treating melanoma in this age group.

## 2. Methods

A retrospective multicenter investigation was performed to obtain information regarding primary cutaneous melanomas diagnosed in patients followed at Humanitas Research Hospital, Rozzano (MI), Italy; Ospedale San Raffaele, Milan (MI), Italy; ASST Papa Giovanni XXIII, Bergamo (BG), Italy; IRCCS IFO San Gallicano, Rome, Italy. To identify AYA melanomas, we conducted a computer-based search of the clinical records of all patients who were registered in our melanoma database between 1 January 2010 and 30 September 2023. Therefore, according to the literature [1], patients with AYA melanoma (≤40 years) were separated from those with non-AYA melanoma (>40 years).

The pathological and clinical information was extracted from electronic databases. The following parameters were registered and are outlined in Table 1 and Table 2: demographics (gender, age), anatomical localization (head/neck, trunk, limbs), histological type of primary melanoma, Breslow thickness (<1 mm or ≥1 mm), presence or absence of ulceration, presence or absence of regression (<40% or ≥40%), number of nevi (≤50 or ≥51), and sentinel lymph node (SLN) biopsy status (positive or negative for metastatic cells), disease-free survival (DFS) and overall survival (OS). We classified melanomas as thin, as described in prior research when their Breslow thickness was <1 mm [10,11].

We followed all patients from the first visit to their death or last follow-up. In accordance with the most recent follow-up guidelines, all patients received routine follow-up in our departments, including dermatological examinations and instrumental assessments using radiography, sonography, CT, MRI, bone scintigraphy, and PET.

## 3. Statistical Analysis

Assuming that the effects of the predictor variables are constant over time, we used Spearmen’s Rank Correlation between the single predictors among AYA and non-AYA melanoma groups at the time of first diagnosis. Subsequently, the independent predictive factors were assessed by multiple logistic regression. Disease-free survival (DFS) was calculated from the diagnosis of the primary tumor to the date of the eventual first metastatic event, while overall survival (OS) was calculated from the first melanoma diagnosis to death and/or last follow-up. The Kaplan–Meier product was used to estimate curves for overall survival, and the log-rank test was used to evaluate differences between the survival curves. Cox proportional hazards-regression was finally performed as further survival analysis. A *p*-value of <0.05 was considered statistically significant. MedCalc software version 23.0.06 (Ostend, Belgium) was used to perform statistical analysis.

## 4. Ethical Consideration

The study followed the guidelines set by the Helsinki Declaration principles, which ensured that the research was done ethically. All patients consented to have their anonymized data used for scientific purposes. Since the study was conducted retrospectively, without deviating from standard clinical practice, it was not necessary to consult the ethics committee for review.

## 5. Results

A total of 1.452 melanoma patients have been included in the current analysis, with a median age of 47 years (ranging between 15 and 94 years), with 756 (52%) male and 696 (48%) female. Among them, 598 patients (41%) showed melanoma ≤40 years (AYA melanomas), and the remaining 854 (58.8%) were non-AYA melanomas (>40 years). We found a female prevalence in the AYA group (60%) versus a male prevalence in the non-AYA group (60%), reaching statistical significance (*p* < 0.0001).

The anatomic site of the primary melanoma was different between the two groups: in the AYA group, a total of 14 (2%) patients showed a head/neck melanoma, 295 (49%) patients in the trunk, and the remaining 289 (48%) in the limbs; conversely, in non-AYA patients, the head/neck was involved in 31 (3.6%) patients, the trunk in 558 (65.3%) patients, and the limbs in the remaining 265 (31%) (*p* < 0.0001). Regarding melanoma histotypes, we found that SSM and in situ melanomas were the most present in AYA (respectively 60% and 36%), followed by spitzoid (2.8%), nodular melanoma (0.8%), and lentigo maligna (0.2%). While analyzing non-AYA melanomas, we found that in situ and SSM melanomas were the most frequent types (47.7% and 47.7%, respectively), followed by lentigo maligna (2.8%), nodular melanoma (1.4%), acral-lentiginous (0.4%), and spitzoid melanoma (0.1%).

Breslow thickness values were reported in 331 cases of AYA melanoma and 425 cases of non-AYA melanoma. In both groups, primary melanomas ≤1.00 mm were the ones most represented, with 87% and 83% of cases, respectively. Finally, we reported the ulceration status in 1.023 cases, revealing no differences between AYA and non-AYA melanoma patients, with ulceration present in 3% and 7% of primary melanomas, respectively. Also, regarding regression, we did not find significant differences between AYA and non-AYA melanoma patients. Regarding the total number of nevi per patient, we did not find any significant differences, with 53.7% of AYA patients and 41.3% of non-AYA melanoma patients having a total number of nevi ≥ 51. Finally, no significant statistical difference in SLN status was present between the two groups, with a positive SLN in 8% of AYA melanoma patients and 21% of non-AYA melanoma patients.

Subsequently, the independent predictive factors were assessed by multiple regression. Therefore, performing multiple logistic regression, the predictor’s gender (*p* < 0.0001) and anatomic site (*p* = 0.001) maintained statistical significance.

**Figure 1:**
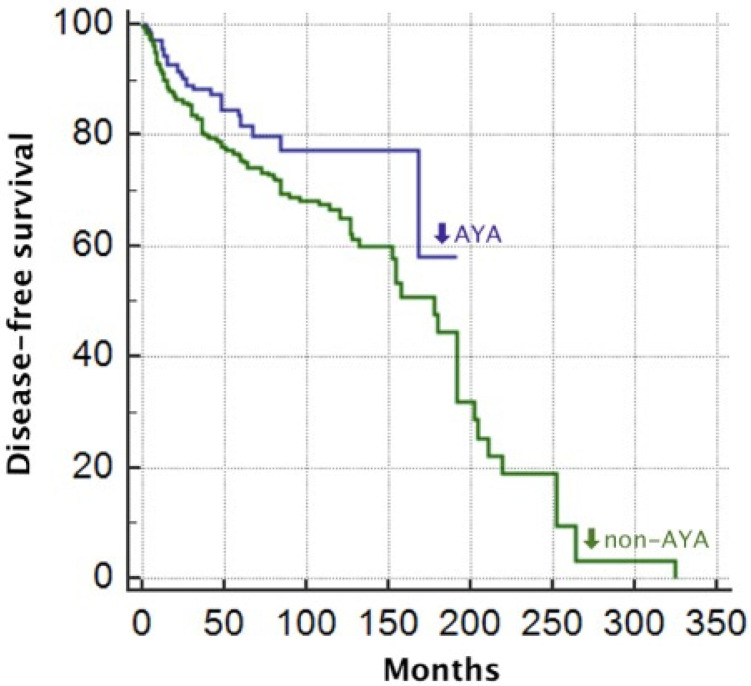
Disease-free survival of Adolescent and Young Adults (AYA) and non-AYA since diagnosis.

The mean DFS of the whole cohort was 154 months, while the mean OS was 324 months. Comparing DFS between the two groups, we found that DFS in AYA patients was higher than in non-AYA patients, with a DFS of 152 months and 149, respectively (*p* = 0.01; Figure 1), while overall survival (OS) was 236 months for AYA patients and 322 months for non-AYA patients, but without reaching the statistical significance (*p* = 0.2) Performing Cox proportional hazards-regression, the statistical significance for DFS was confirmed (*p* = 0.008), unlike OS which did not reach significance (*p* = 0.2).

## 6. Discussion

Melanoma is one of the most common malignancies causing morbidity and mortality in the United States and Europe. Specifically, it is the fifth most common cancer among male patients and the seventh most common malignancy among women [2]. Besides, after breast cancer, among young adults, melanoma is the most common malignancy. According to the National Cancer Institute recently reported from the Surveillance, Epidemiology, and End Results, from 1973 to 2004, the age-adjusted annual incidence of melanoma among teen patients and young adults between 19 years and 39 years increased respectively from 4.7 cases per 100,000 persons to 7.7 cases per 100,000 persons [2,12]. The increase in melanoma cases among young people can be attributed to both incorrect habitual behaviors (e.g., UV exposure, tanning beds, incorrect use of sunscreens) and advancements in non-invasive dermatological diagnostic techniques. Indeed, in the last decades, innovations in non-invasive dermatological technologies, such as dermoscopy, in vivo reflectance confocal laser microscopy (RCM), and Line-field Confocal Optical Coherence Tomography (LC-OCT), have greatly enhanced the ability to identify skin cancers at an earlier stage. These tools allow for more precise examination of suspicious lesions, increasing the likelihood of detecting melanoma in its initial stages, particularly among young individuals who might not have been diagnosed as early in the past. Consequently, while more cases are being identified, this trend also reflects the positive impact of technological advancements that support earlier diagnosis and better management of skin cancer.

### Clinical Implications

In our analysis, there is a significant difference in demographic distribution between the two groups, with females being more prevalent in AYA and males in the non-AYA group. This finding is consistent with the available literature [1,13,14]. Early menarche, coupled with childhood UV exposure, likely contributes to heightened endogenous estrogen levels, potentially explaining the elevated incidence of melanoma in young females compared to males [15]. In this regard, in 1996, a survey by phone carried out by the American Academy of Dermatology revealed that even with a rise in public awareness about the dangers of UV light exposure, the occurrence of severe sunburns and the use of tanning beds had gone up, particularly among women [2,16,17,18,19]. Besides, a recent telephone survey indicates that young women are significantly more likely than young men to engage in indoor tanning [2]. Additionally, UV exposure during adulthood, as well as tanning bed use and sunburns during childhood and adolescence, may contribute to the development of melanoma [2]. However, at the same time, preventive care is more frequent in women than in men, with a higher probability of diagnosing melanoma in women than in men [16]. This increased vigilance in women may be attributed to a higher level of awareness about skin health, a greater concern about aesthetic appearance, and more frequent interactions with healthcare professionals for routine care, which provide more opportunities to discuss skin health. Contrariwise, men are generally less likely to perform regular skin self-examinations or seek professional skin checks, potentially due to lower awareness of skin cancer risks or cultural attitudes that discourage concern over skin health.

Regarding age, interestingly, the median age of our population was 47 years, which is lower than the general melanoma median age, which usually ranges between 50 years and 65 years [20]. Increased awareness and access to screening programs in our study area may have contributed to earlier diagnoses. Besides, this observation confirms the fact that there is a notable trend of decreasing average age of onset of melanoma [21], driven by behavioral, genetic, and environmental factors, as well as increased awareness of screening among patients. Certainly, the genetic differences between juvenile melanomas and adult melanomas (BRAF more frequent in the former, NRAS, NF-1 and TP53 in the latter) associated with differences in the anatomical localizations of primary melanomas (intermittent sun-exposed areas in juvenile melanomas and chronically sun-exposed areas in adult and elderly patients) conceal physio-pathological mechanisms that could also be relevant for the setting of a different follow-up and therapeutic approach in the future. An important cut-off for AYAs melanoma is to evaluate patients ≤10 years versus patients >10 years, respectively, prepubertal from post-pubertal patients. According to recent studies [22,23,24,25], nevus-associated melanoma is higher among patients ≤10 years, and Spitzoid melanoma is also more common in patients 10 years of age, presenting with a higher Breslow thickness and a higher propensity for nodal involvement but usually showing a more indolent biological behavior and a better prognosis [22].

In our study group, a large proportion of melanoma is in situ melanoma (Table 1). However, superficial spreading melanoma remains the most common histological type in both groups; specifically, SSM was more common in AYA than non-AYA (60% vs. 47.7%). A study by van der Kooij et al. found similar results, observing a statistically significant prevalence of SSM in AYA compared to adults [14]. Most likely, the increased presence of SSM in our samples reflects the general higher incidence of SSM among all melanoma histotypes in the general population, accounting for about 60% of all melanoma cases. There was a notable distinction in localization between the two cohorts: trunk and limb involvement were nearly equivalent for AYA melanoma (49% and 48%), whereas non-AYA melanomas were considerably more prevalent in the trunk (65.3%). Van der Kooij, Monique K. et al., and Livestro D.P. et al. have both reported similar findings [5,14]. This disparity may indicate the impact of age-related changes in sun exposure and skin protection habits. The data from Caroppo et al. [15], which show a higher incidence of the trunk among the AYA group, contrasts with this data, raising questions about the significance of lifestyle habits and socio-cultural context. These include the use of tanning beds and engaging in outdoor activities, both of which can differ significantly among various demographic groups.

Breslow thickness was reported to be <1.00 mm, with a similar percentage in both groups. However, other studies [3,14,26] reported a lower Breslow level in AYA melanoma compared to non-AYA melanoma, suggesting a potential for more invasive melanomas in this age group. We observed the ulceration status in both groups, but it was relatively uncommon (3% in AYA and 7% in non-AYA). This is consistent with the current literature [4,5].

We did not find any statistically significant difference in the presence of regression, total number of nevi, or SLN status between the two groups. The existing literature lacks enough information regarding the disparity in regression status between the AYA and non-AYA groups.

While regarding SLN status, a study found that AYA patients were more likely than non-AYA patients to have an SLN biopsy performed [4], Del Fiore et al. found that the prevalence of positive SLN was comparable in the AYA and non-AYA groups [3].

Regarding survival analysis, we found a higher DFS in AYA (152 months) compared to non-AYA (149 months), confirming the data from the literature [3], although in contrast to some reports that found a worse DFS in AYA patients [5,6]. Most likely, the better DFS observed in AYA patients may be related to the fact that often young patients usually have fewer comorbidities and a stronger overall health status, as well as young patients tend to have a more robust immune system. All of these considerations could impact the DFS we observed in our case series.

## 7. Limitation

This study has several limitations, such as its retrospective nature and the absence of key clinical and pathological data (e.g., sun exposure and familial history) for some patients. The missing data may have introduced bias and affected the robustness of the analyses. Finally, the absence of patients under 10 years of age did not allow for a stratified analysis for this cutoff.

## 8. Conclusions

Melanoma is the second most common malignancy diagnosed in young adults each year, therefore, identifying the main clinical-pathological characteristics is critical to best following this class of patients, who currently have no follow-up or specific therapies but are monitored and treated as non-AYA patients. Regrettably, the literature still underrepresents this type of melanoma. Although we have identified some recurrent features in our case series and the literature, the heterogeneity of this pathology does not allow us to highlight any specific characteristics, confirming the contradictory results currently present in the literature on AYA melanomas. Further extensive investigations, incorporating genetic analyses and involving multiple centers, have the potential to advance our understanding of this interesting phenotype of melanoma in the future.

## Figures and Tables

**Table 1:** This table shows the clinicopathological baseline analyzed in our study.

	*n* ^AYA^	*n* ^non-AYA^	% ^AYA^	% ^non-AYA^	*p* *	*p* **
**Gender**					<0.001	<0.0001
Male	239	342	40	60		
Female	359	512	60	40		
**Anatomic Site**					<0.0001	0.0001
Head/Neck	14	31	2	4		
Trunk	295	558	49	65		
Limbs	289	265	48	31		
**Histological Type**					NS	NS
In Situ	215	407	36	47.7		
SSM	360	407	60.2	47.7		
Nodular	5	12	0.8	1.4		
ALM	0	3	0	0.4		
Spitzoid	17	1	2.8	0.1		
LM	1	24	0.2	2.8		
**BRESLOW ^≠^**					NS	NS
<1.00	289	355	87	83		
≥1.00	42	70	13	17		
**Ulceration ^∞^**					NS	NS
Presence	5	57	3	7		
Absence	177	784	97	93		
**Regression**					NS	NS
Presence	242	407	60	57		
Absence	159	305	40	43		
**Number of NEVI**					NS	NS
≤50	31	215	46.3	58.7		
≥51	36	151	53.7	41.3		
**SLN**					NS	NS
Positive	6	28	8	21		
Negative	68	106	92	79		

* Spearman’s Rank correlation; ALM: acral-lentiginous melanoma; LM: lentigo maligna; NS: non significant; ^≠^ among Adolescent and Young Adults (AYA) melanoma patients, the Breslow value was reported in 331 cases, while Breslow thickness was reported in 425 non-AYA melanoma patients; ^∞^ the status of ulceration was present in 1.023 cases; Regression status (presence/absence) was reported in 411 AYA melanoma patients and 712 non-AYA melanoma patients; SLN means sentinel lymph node biopsy; ** multiple regression analysis performed with multiple logistic regression.

**Table 2:** This table shows the mean disease-free survival (DFS) and overall survival (OS) between Adolescent and Young Adults (AYA) and non-AYA patients. NS = Non-Significant.

	AYA	Non-AYA	*p*
DFS	152	149	0.01
OS	236	322	NS

## Data Availability

The raw data supporting the conclusions of this article will be made available by the authors on request.
